# Another Reason for Using Caffeine in Dermocosmetics: Sunscreen Adjuvant

**DOI:** 10.3389/fphys.2019.00519

**Published:** 2019-05-03

**Authors:** Catarina Rosado, Viviane Kaori Tokunaga, Rafael Sauce, Camila Areias de Oliveira, Fernanda Daud Sarruf, Roberto Parise-Filho, Elisabete Maurício, Tânia Santos de Almeida, Maria Valéria Robles Velasco, André Rolim Baby

**Affiliations:** ^1^CBIOS – Research Center for Biosciences and Health Technologies, Universidade Lusófona, Lisbon, Portugal; ^2^Department of Pharmacy, School of Pharmaceutical Sciences, University of São Paulo, São Paulo, Brazil; ^3^IPclin, Pesquisa Integrada, Clinical Research Company, São Paulo, Brazil

**Keywords:** caffeine, sunscreen, sun protection factor, UV radiation, cutaneous compatibility

## Abstract

The excessive exposure to ultraviolet (UV) radiation is the main cause of skin cancer, the most commonly diagnosed cancer in the world. In this context, the development of innovative and more effective sunscreens, with bioactive compounds like caffeine, displaying antioxidant and anticancer potential, is required. This research work assessed *in vitro* and *in vivo* the efficacy and safety of topical sunscreen formulations containing caffeine as an adjuvant of the UV filters. Sunscreens were prepared with 2.5% w/w caffeine or in the absence of this compound. In order to evaluate the safety of these formulations, *stratum corneum* hydration, skin barrier and colorimetry were assessed *in vivo* in healthy subjects before and after skin treatment with the samples. The efficacy of the sunscreens was assessed *in vitro*, using PMMA plates and a spectrophotometer equipped with an integrating sphere; and *in vivo* by the determination of the sun protection factor (SPF). None of the formulations caused erythema or impaired the skin barrier function. The *in vitro* functional characterization showed higher SPF values for the caffeine formulation. The *in vivo* studies also confirmed the higher SPF value of the formulation combining caffeine with the filters, compared to the caffeine-free sample. This improvement contributed to an increase of, approximately, 25% in the *in vivo* anti-UVB protection. In conclusion, caffeine was well tolerated by the skin and increased the photoprotective activity, being a new alternative adjuvant in sunscreens formulation.

## Introduction

**FIGURE 1:**
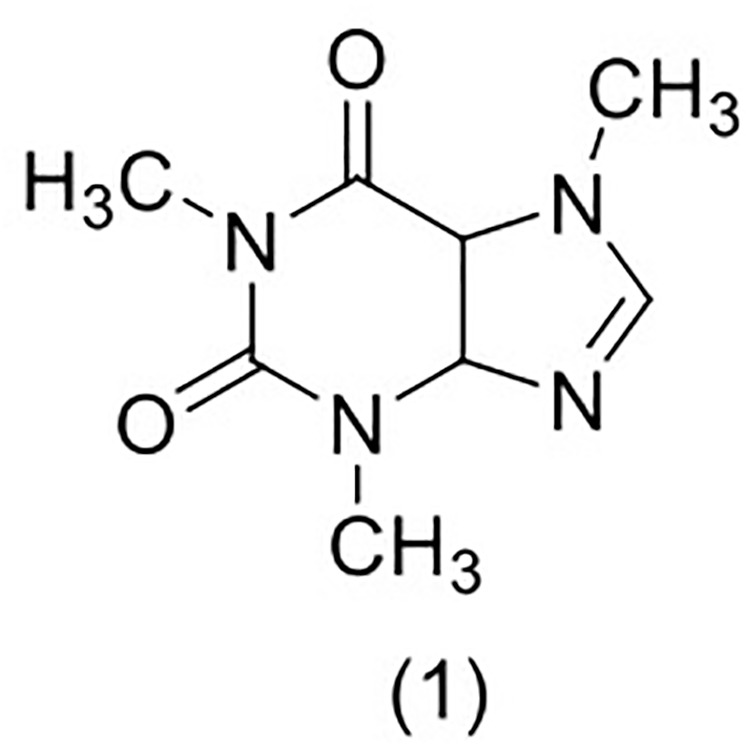
Chemical structure of caffeine (1).

The excessive exposure to ultraviolet (UV) radiation has led to an increase in skin cancer, the most common type of cancer diagnosed in the world (International Agency for Research on Cancer [IARC], 2012; American Cancer Society, 2016), thus increasing the relevance of the development of advanced and more effective photoprotective formulations. The original sunscreens only aimed to absorb or divert the radiation through the use of chemical or physical UV filters, but the current formulation trend is to provide a more complete protection by using bioactive compounds with beneficial properties (Cestari et al., 2012; Peres et al., 2016, 2017). Caffeine (1,3,7-trimethylpurine-2,6-dione) is a substance of the methylxanthine class (Figure 1), found especially in coffee, one of the world’s most commonly consumed beverages (Martini et al., 2016). This substance has been used in pharmaceutical and cosmetic preparations for a long time because of its beneficial effects on the skin, namely, anti-cellulite and anti-aging (Rodrigues et al., 2016). More recently, caffeine has additionally been studied for its antioxidant and anticancer effects (Shi et al., 1991; Devasagayam et al., 1996; Lu et al., 2007; Kawasumi et al., 2011), but it also has shown photoprotective properties in an animal model and, thus, exhibits a high potential to function as a sunscreen adjuvant (Lu et al., 2007).

Innovative sunscreens aim to fully protect the cutaneous tissue from the deleterious effects of UV radiation, which is considered an important risk factor associated with skin cancer (International Agency for Research on Cancer [IARC], 2012; American Cancer Society, 2016). This disease is characterized by the uncontrolled growth of abnormal skin cells that occurs when there is damage to DNA, triggering mutations or genetic defects that lead the cells to rapidly multiply and form malignant tumors (Skin Cancer Foundation, 2016). Caffeine has shown to be a potential anticancer bioactive molecule, causing apoptosis in preneoplastic cells and destruction of cells with damaged DNA (Lu et al., 2002; Lu et al., 2005; Kawasumi et al., 2011).

Additionally, exposure to UV radiation causes oxidative stress, which is also associated with skin cancer (Chen et al., 2012) by generating free radicals that damage proteins, DNA, RNA, sugar molecules, and lipids (Lu et al., 2010; Craft et al., 2012). Oral and topical applications of antioxidants, in combination with sunscreens, have shown to enhance skin photoprotection (Masnec et al., 2010). Therefore, it is likely that sunscreen products containing antioxidants could more significantly reduce skin damage, as well as skin cancer rating. Caffeine has been reported to scavenge highly reactive free radicals and to defend crucial biological molecules against these species (Devasagayam et al., 1996). Furthermore, the work of Szeto and Tong (2010) showed that caffeine at 5.0 μmol/L reduced 5-methoxypsoralen-induced phototoxicity with UVC exposure. Caffeine has been used extensively as a cosmetic ingredient not only because of its bioactivity, but also due to its low toxicity profile (Nawrot et al., 2003; Doepker et al., 2016) and numerous works can be found in the literature reporting caffeine skin permeation (Zesch et al., 1979; Dias et al., 1999; Johnson et al., 2006; Herman and Herman, 2013). In a multi-center comparison study, the caffeine mean maximal flux was found to be 2.24 μg/cm^2^/h (van de Sandt et al., 2002).

Considering the exposed, our research work aimed to assess *in vitro* and *in vivo* the cutaneous effects of formulations containing caffeine as an adjuvant of UV filters, in order to further establish its safety and efficacy as a functional ingredient of sunscreens.

## Materials and Methods

### Formulations

The efficacy and safety of caffeine was evaluated in association with three UV filters: ethylhexyl methoxycinnamate (7.5%), avobenzone (3.0%) and titanium dioxide (5.0%). Oil-in-water emulsions were prepared in the absence of caffeine or containing this bioactive compound at 2.5% w/w, as shown in Table 1. The dermocosmetic vehicle contained the following ingredients (% w/w): glycerin (5.0%); ammonium acryloyldimethyltaurate/VP copolymer (1.5%); disodium EDTA (0.2%); sodium benzoate (2.5%); isopropyl myristate (5.0%); butylated hydroxytoluene (0.05%); cetearyl alcohol/dicetyl phosphate/ceteth-10 phosphate (7.0%); dibutyl adipate (3.5%); imidazolidinyl urea (1.0%); and purified water (enough to complete the 100.0% of the vehicle). All solvents and ingredients were of cosmetic, pharmaceutical or analytical grade and were used as received, without any further purification.

**Table 1:** Active ingredient composition (% w/w) of sunscreens.

Composition^a^	Concentration (% w/w) Formulation	
	Caffeine free	2.5% Caffeine
**Ethylhexyl methoxycinnamate**	7.5	7.5
**Avobenzone**	3.0	3.0
**Titanium dioxide**	5.0	5.0
**Caffeine**	–	2.5

*^a^Qualitative composition was reported in accordance with INCI (International Nomenclature of Cosmetic Ingredient).*

### *In vivo* Skin Compatibility Assay

Twelve healthy male and female volunteers participated in the study, after oral information and written consent. This procedure was performed in accordance with the Helsinki Declaration and with the ethical standards of the local responsible committee on human experimentation. Three sites were marked in the volar forearm of the volunteers. Epicutaneous patches (Finn Chambers^®^, Epitest) containing either 2.5% w/w caffeine sunscreen formulation, caffeine-free sunscreen formulation, or filter paper disk soaked in distilled water (negative control), were applied at each site for 24 h.

*Stratum corneum* (SC) hydration was evaluated with a Corneometer^®^ CM825 (CK Electronics GmbH), and skin barrier function was probed through the transepidermal water loss (TEWL) measured by a Tewameter^®^ TM 300 (CK Electronics GmbH), according to the guidelines of Pinnagoda et al. (1990). Skin color was non-invasively measured by colorimetry, using a Minolta^®^ Chroma Meter CR-300 (Minolta Camera). The parameter *a*^∗^ provided by the device reflects the red chromaticity and can be used to quantify an increase in erythema (Oliveira et al., 2016). All measurements were performed in triplicate, and the CIE Lab system was used (Piérard, 1998). The basal values were determined before patch application, and further measurements were made at 24 h, 2 h after patch removal (Oliveira et al., 2015). To minimize the effect of inter-individual variability, the results were analyzed as the ratio between the values obtained after patch application and the basal values (Oliveira et al., 2015).

### *In vitro* Functional Characterization of the Sunscreen Formulations

Functional characterization was performed in a spectrophotometer equipped with an integrating sphere (Labsphere^®^ UV-2000S Ultraviolet Transmittance Analyzer). The formulations were weighed (0.75 mg/cm^2^) and uniformly applied over polymethylmethacrylate (PMMA) plates, mimicking the rough skin surface. Samples were arranged in a suitable container covered with quartz plate, to avoid ambient interference, and thus exposed to the sunlight. The exposure of the formulations to solar radiation was conducted between late April and early June of 2014, for 2 h (11 a.m. to 1 p.m.), in São Paulo city, SP, Brazil, with an estimated irradiation intensity of 151.75 mW/m^2^ (Satellite Division and Environmental Systems [DSA], 2014). Replicas of three (nine measurements per PMMA plate) were performed for each sample; one previously to the solar irradiation (t_0_) and one after the solar stress (t_f_), being measured after 120 min (Lu et al., 2007).

The records of the spectrophotometric values were performed in the wavelength range between 250 and 450 nm with a progression rate of 1.0 nm (Food and Drug Administration [FDA], 2011). The estimated sun protection factor (SPF), critical wavelength (nm) and photostability were determined using the UV-2000^®^ software (Velasco et al., 2008; Cosmetics Europe, 2011; Dario et al., 2018; Tomazelli et al., 2018).

### *In vivo* Determination of SPF

The *in vivo* SPF assessment was performed in accordance with the ethical standards of the local responsible committee on human experimentation, with the Helsinki Declaration and according to the International SPF Test Method (Cosmetics Europe, 2006). The test involved 10 healthy male and female volunteers with skin Fitzpatrick types II and III, after oral information and written consent; samples were applied at 2.0 mg/cm^2^; the Multiport^®^ 601 (Solar Light Company) solar UV simulator was used. Studies were conducted by IPclin (Jundiaí, SP, Brazil) (Oliveira et al., 2016; Tomazelli et al., 2018). SPF value (Eq. 1) was defined as the UV energy required to produce a minimum erythemal dose (MED), or redness, on protected skin divided by the UV energy required to produce a MED on unprotected skin (Peres et al., 2018):

(1) $$\mathrm{SPF}=\frac{\mathrm{MED of protected skin}}{\mathrm{MED of unprotected skin}}$$

### Statistical Analysis

Statistical analysis of the data was performed using one-way ANOVA test followed by the Tukey test for multiple comparisons. The GraphPad Prism^®^ (Version 7) software was used, with a significance level of 5.0% (α = 0.05).

## Results

### *In vivo* Skin Compatibility Assay

**FIGURE 2:**
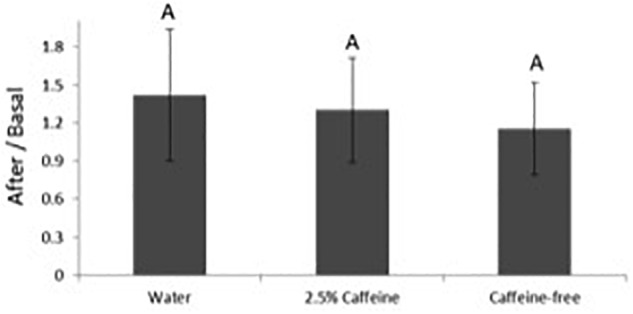
Variation in *stratum corneum* (SC) hydration before and after application of the different formulations. The results were analyzed as the ratio between the values obtained after patch application over the basal values. Different letters for the same parameter indicate statistically significant differences between samples (*p* < 0.05; mean + SD, *n* = 12).

**FIGURE 3:**
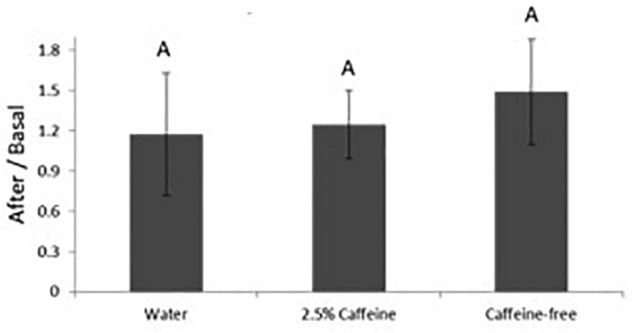
Variation in transepidermal water loss before and after application of the different formulations. The results were analyzed as the ratio between the values obtained after patch application over the basal values. Different letters for the same parameter indicate statistically significant differences between samples (*p* < 0.05; mean + SD, *n* = 12).

**FIGURE 4:**
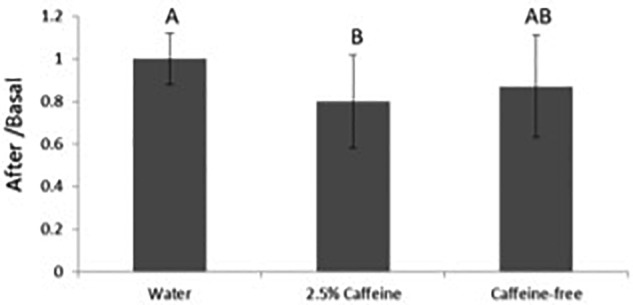
Variation in skin redness before and after application of the different formulations. The results were analyzed as the ratio between the values obtained after patch application over the basal values. Different letters for the same parameter indicate statistically significant differences between samples (*p* < 0.05; mean + SD, *n* = 12).

Results were analyzed as the ratio between the values obtained in each site after patch application and its respective basal values, thus decreasing the impact of inter-individual variability. Although a slight increase in SC hydration was observed, probably attributable to occlusion or to the emollients in the O/W emulsion, no differences were established among the sites treated with the formulations and the negative control (Figure 2). No significant impact on the TEWL and, therefore, in the skin barrier function was observed in any of the sites (Figure 3). The colorimetry assay was employed to further ascertain the cutaneous compatibility of the formulations. A significant decrease in the skin redness was detected after treatment with the two formulations in comparison with the negative control, indicating that the formulations were well-tolerated and did not cause erythema (Figure 4).

### *In vitro* Functional Characterization of the Formulations

The functional characterization was analyzed in the absence (caffeine-free) and at 2.5% w/w caffeine, in different solar irradiation times (t_0_ and t_f_) (Table 2). According to this data, it was observed that irradiation, in function of the time of exposure, affected positively the SPF values of both caffeine-free and 2.5% caffeine samples, but the latter provided significantly higher SPF values.

**Table 2:** *In vitro* SPF and critical wavelength (nm) values (mean and standard deviation) as a function of irradiation time.

	Caffeine-free		2.5% of caffeine	
Irradiation time	SPF	Critical wavelength (nm)	SPF	critical wavelength (nm)
**t_0_**	29.0 ± 0.0**^A^**	378 ± 0.0**^A^**	38.5 ± 2.1**^A^**	379 ± 0.0**^A^**
**t_f_**	36.0 ± 0.0**^B^**	377 ± 0.0**^B^**	51.0 ± 0.0**^B^**	378 ± 0.0**^B^**

*Different letters for the same parameter indicate statistically significant differences between samples (p < 0.05 and n = 3).*

### *In vivo* Determination of SPF

The value measured in the 2.5% caffeine formulation, with SPF of 19.34, was statistically significantly higher when compared to the caffeine-free sample, with SPF 15.43 (Table 3).

**Table 3:** *In vivo* SPF.

Sample	*In vivo* SPF
Caffeine-free	15.49 ± 0.39**^A^**
2.5% Caffeine	19.34 ± 0.34**^B^**

*Different letters for the same parameter indicate statistically significant differences between samples (p < 0.05 and n = 10).*

## Discussion

The sunscreen formulation containing caffeine at 2.5% w/w presented a higher SPF value on both *in vitro* and *in vivo* tests when compared to the caffeine-free sample. This improvement contributed to an increase of, approximately, 25% in the *in vivo* anti-UVB protection. In addition, the *in vivo* skin compatibility assays showed that the formulation with caffeine did not cause a negative impact in SC hydration or in skin barrier function, nor did they cause any erythema, even when applied under occlusion for 24 h.

Caffeine has been widely studied for its potential beneficial actions in the human organism. Shi et al. (1991) reported that caffeine effectively scavenged the hydroxyl radical by using an electron spin resonance spin trapping mechanism. Devasagayam et al. (1996) showed its antioxidant effect by inhibition of lipid peroxidation of rat liver microsomes induced by hydroxyl radical, peroxyl radical and singlet oxygen. These authors registered that the caffeine antioxidant activity was similar to glutathione and that it was higher than ascorbic acid^7^. Other studies have shown the anticancer effects of caffeine. It was demonstrated that the topical application of caffeine possibly caused apoptosis in preneoplastic cells (Lu et al., 2002, 2005). Kawasumi et al. (2011) researched the protection of UV-induced skin carcinogenesis on SKH-1 hairless mice using topical application of caffeine, showing that this compound promoted the activity of the ATR gene, involved in the destruction of cells with damaged DNA, consequently more likely to become cancerous cells. Moreover, caffeine has shown to inhibit UVB-induced carcinogenesis (Lu et al., 2007). However, to date, there is no data in the specialized literature that proves its photoprotective effect by the SPF establishment.

In our study, caffeine alone did not provide a significant SPF value (data not shown), Nevertheless, when in association with the sunscreen system, containing two chemical UV filters - ethylhexyl methoxycinnamate and avobenzone - and the physical filter titanium dioxide, it acted as a SPF enhancer. This improvement can probably be attributed to an antioxidant or an anti-inflammatory activity, attenuating the erythema caused by the UV radiation or even, delaying the erythema formation. Additionally, caffeine contributed as a photostabilizer for the chemical filters, which are known, when associated, to undergo an irreversible photochemical reaction leading to loss of UV protection (Dondi et al., 2006). Other studies conducted by our group with antioxidants, such as rutin (Tomazelli et al., 2018) and ferulic acid (Peres et al., 2018), have shown to be similarly advantageous in sunscreen formulations.

According to the specialized literature, caffeine absorbs in the spectral range between 244 and 295 nm in water, with λ at 272.8 nm (Atomssa and Gholap, 2011). Also, its molar absorption coefficient (max ε) in water, which is how strongly a substance absorbs radiation of a given frequency, is 920 ± 0.85 m^2^mol^-1^, possibly not being enough to develop on its own a substantial SPF (Atomssa and Gholap, 2011). However, it should be noted that the SPF value of 2.5% w/w caffeine associated with the UV filters, after the sunlight exposure, was significantly higher than the one measured before this stress condition, and provided a much higher increase that the one observed in the caffeine-free formulation. The main role of organic molecules in sunscreens (UV filters), most of which contain aromatic rings conjugated with carbonyl groups, is to absorb UVA and/or UVB radiation and convert the electronic excitation into, for example, vibrational energy, without modifying themselves (Karsili et al., 2014; Tan et al., 2014). However, in a photochemical organic reaction, molecule alterations may occur, like electrocyclic reactions, radical reactions, photoisomerization, and Norrish reactions, which could change the photoprotective propriety of the system (Klan and Wirz, 2009; Turro et al., 2010). Horbury et al. (2016) showed that the photoexcitation of ferulic acid and caffeic acid resulted in an isomerization pathway, which the *cis*-isomer provides a comparable level of photoprotection as the *trans*-. Karsili et al. (2014) described that adding the hydroxy/methoxy groups to the ring of molecules, like cinnamic acid, results in red-shifting (increasing in wavelength) the UV absorption spectrum, matching better for sunscreen applications. Considering the exposed, it is plausible to infer that the caffeine molecule could pass through modifications when electronically excited, developing a better sunscreen activity by SPF enhancement.

**FIGURE 5:**
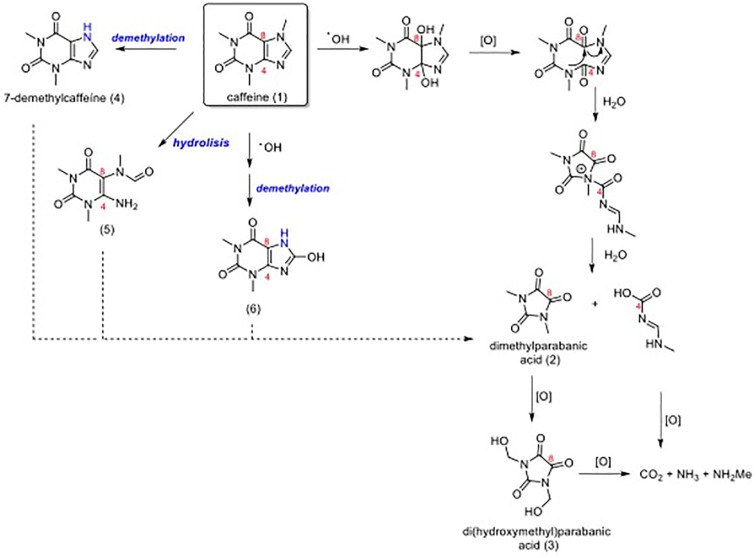
Degradation reaction of caffeine (1). The photolytic degradation mechanism of caffeine (1) to dimethylparabanic acid (2) involves the hydroxy radical attack on caffeine’s double bond D^4,8^. Hydroxylation and subsequent oxidation processes generate compounds 2 and 3, which the substances CO_2_, NH_3_, and NH_2_Me are formed more slowly. Noteworthy, the oxidation products 4, 5, and 6 may also be converted into dimethylparabanic acid (2) via a similar mechanism.

Caffeine has been reported (Devasagayam and Kesavan, 1996; Horbury et al., 2016) to scavenge highly reactive free radicals, including hydroxyl radicals and excited states of oxygen, and to protect crucial biological molecules against these species. Also, studies indicated that caffeine degraded slowly by direct photolysis (>170 h in artificial sunlight) (Jacobs et al., 2012). Moreover, experimental and *in silico* studies carried out by Dalmázio et al. (2005) and León-Carmona and Galano (2011a), respectively, have reported that caffeine has the ability to capture free radicals and undergo subsequent degradation processes, especially by radical formation of adducts which can generate species that cause wavelength-shifting. The authors have determined caffeine degradation by UV/TiO_2_ system, which is known to produce *in situ* hydroxyl-radical species (Dalmázio et al., 2005). The proposed mechanism involves successive steps of hydroxylation and oxidation to generate compounds such as CO_2_, H_2_O and NH_3,_ and NH_2_Me (Figure 5). According to Dalmázio et al. (2005), the results indicated that, although caffeine was almost completely consumed, mineralization (CO_2_, H_2_O, and NH_3_ formation) occurred slower under oxidative conditions in the presence of UV/TiO_2_. Noteworthy, in other systems studied, the authors have achieved very similar results in the identification of species formed by caffeine degradation. Therefore, degradation of caffeine is likely to generate persistent organic intermediates that are not so efficiently oxidized as compared to caffeine and this could be the explanation to increased photoprotection.

**FIGURE 6:**
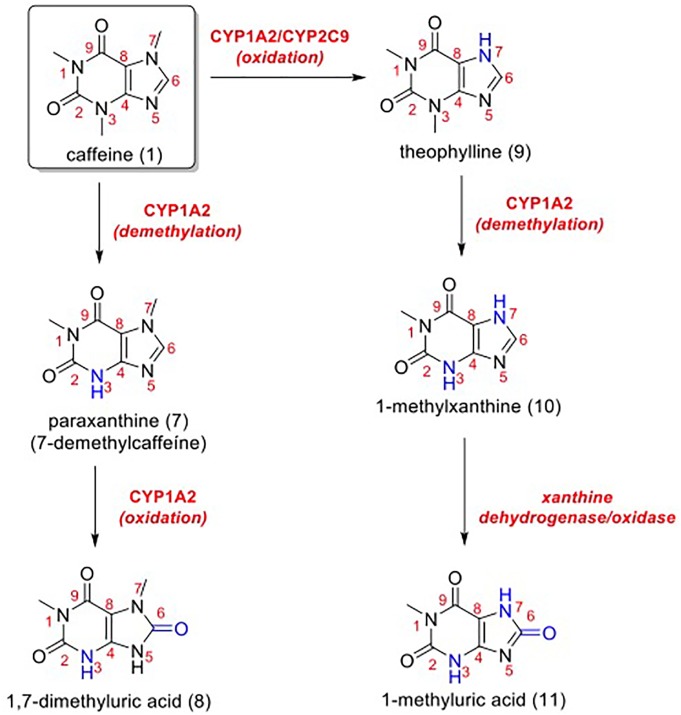
Metabolic pathways for caffeine (1). In blue, positions that suffered metabolic reactions.

One other hypothesis is that caffeine (1) could be metabolized when topically applied on the human cutaneous tissue, and these metabolites might be related to the enhanced photoprotection obtained *in vivo* and to beneficial actions (Figure 6). Caffeine is mainly metabolized by CYP1A2 in the liver, and this enzyme is also present in our skin at low concentrations, which could lead to the formation of paraxanthine (7) (Hakooz, 2009; Luu-The et al., 2009; Hu et al., 2010). Paraxanthine (7) may be once more metabolized into 1,7-dimethyluric acid (8) by CYP1A2 (Hakooz, 2009). Another important metabolite pathway that could happen in the skin is the metabolization of caffeine (1) by CYP1A2 or by CYP2C9 (likewise present in the skin at low concentrations), generating theophylline (9) (Hakooz, 2009; Luu-The et al., 2009; Hu et al., 2010). Theophylline (9) is also metabolized by CYP1A2, generating 1-methylxanthine (10), which is then metabolized by xanthine dehydrogenase/oxidase (present in epidermal keratinocytes), forming 1-methyluric acid (11) (Shalbaf et al., 2008; Hakooz, 2009).

Scurachio et al. (2016) demonstrated that 1,7-dimethyluric acid (10) and 1-methyluric acid (11) were efficient as quenchers of triplet riboflavin (which causes oxidative damage to proteins, vitamins, lipids and sterols upon UV absorption) using transient absorption laser flash photolysis. Compound 11 is one of the main metabolic products of caffeine (1) in humans, and it has been reported to act as an antioxidant (Lee, 2000). This molecule is predicted to moderately protect lipids against peroxyl oxidation, being a better radical scavenger than its precursor, caffeine (León-Carmona and Galano, 2011b). Moreover, León-Carmona and Galano (2011a,b) suggested that the antiradical activity of caffeine might be explained more by the action of its own metabolites, rather than by its direct activity. Thus, the significant antioxidant activity of (11) was illustrated by oxygen-radical absorbing capacity (ORAC) and inhibition of LDL peroxidation (Lee, 2000).

## Conclusion

In conclusion, caffeine and some of its metabolites could be acting beneficially in the photoprotection and antioxidant activities, besides a relevant photostabilization profile. In this research work, the interest of combining caffeine with UV filters in a sunscreen formulation was established, and its cutaneous biocompatibility was also confirmed. However, a phototoxicity assay would be indispensable to fully assess the safety of this type of ingredient. Higher SPF was obtained when caffeine was combined with the UV filters ethylhexyl methoxycinnamate, avobenzone and titanium dioxide. Caffeine seemed to be, thus, an interesting bioactive compound for use in sunscreens, acting in synergy as a photoprotector and a photostabilizer.

## Ethics Statement

This study was carried out in accordance with the recommendations of international standards and International Sun Protection Factor Test Method, (Cosmetics Europe, 2006). The protocol was approved by the (CEP-FCF-USP and CBIOS). All subjects gave written informed consent in accordance with the Declaration of Helsinki.

## Author Contributions

AB and CR conceived and designed the experiments. VT, CR, FS, and CdO performed the experiments. CdO, CR, RS, RP-F, and AB analyzed the data. CR, TdA, EM, CdO, RS, RP-F, MV, and AB wrote the manuscript.

## Conflict of Interest Statement

FS was employed by IPclin. The remaining authors declare that the research was conducted in the absence of any commercial or financial relationships that could be construed as a potential conflict of interest.

## References

[B1] American Cancer Society (2016). Available at: http://www.cancer.org/cancer/skincancer/index (accessed April 01, 2017).

[B2] Atomssa T. Gholap A. V. (2011). Characterization of caffeine and determination of caffeine in tea leaves using uv-visible spectrometer. *Afr. J. Pure Appl. Chem.* 5 1–8.

[B3] Cestari T. F. Oliveira F. B. Boza J. C. (2012). Considerations on photoprotection and skin disorders. *Ann. Dermatol. Venereol.* 139 135–143. 10.1016/S0151-9638(12)70116-3 23522628

[B4] Chen L. Hu J. Y. Wang S. Q. (2012). The role of antioxidants in photoprotection: a critical review. *J. Am. Acad. Dermatol.* 67 1013–1024. 10.1016/j.jaad.2012.02.009 22406231

[B5] Cosmetics Europe (2006). *COLIPA Guidelines. Methods for Testing Efficacy of Sunscreen Products.* Brussels: Cosmetics Europe.

[B6] Cosmetics Europe (2011). *COLIPA Guidelines In vitro Method for the Determination of the UVA Protection factor and “Critical Wavelength” values of Sunscreen Product.* Brussels: Cosmetics Europe.

[B7] Craft B. D. Kerrihard A. L. Amarowicz R. Pegg R. B. (2012). Phenol-based antioxidants and the in vitro methods used for their assessment. *Compr. Rev. Food Sci. Food Saf.* 11 148–173. 10.1111/j.1541-4337.2011.00173.x

[B8] Dalmázio I. Santos L. S. Lopes R. P. Eberlin M. N. Augusti R. (2005). Advanced oxidation of caffeine in water: on-line and real-time monitoring by electrospray ionization mass spectrometry. *Environ. Sci. Technol.* 39 5982–5988. 10.1021/es047985v 16173554

[B9] Dario M. F. Oliveira F. F. Marins D. S. S. Baby A. R. Velasco M. V. R. Löbenberg R. (2018). Synergistic photoprotective activity of nanocarrier containing oil of *Acrocomia aculeata* (Jacq.) Lodd. Ex. Martius - Arecaceae. *Ind. Crops Prod.* 112 305–312. 10.1016/j.indcrop.2017.12.021

[B10] Devasagayam T. P. Kamat J. P. Mohan H. Kesavan P. C. (1996). Caffeine as an antioxidant: inhibition of lipid peroxidation induced by reactive oxygen species. *Biochim. Biophys. Acta* 1282 63–70. 10.1016/0005-2736(96)00040-5 8679661

[B11] Devasagayam, P. C. (1996). Radioprotective and antioxidant action of caffeine: mechanistic considerations. *Indian J. Exp. Biol.* 34 291–297. 8698415

[B12] Dias M. Farinha A. Faustino E. Hadgraft J. Pais J. Toscano C. (1999). Topical delivery of caffeine from some commercial formulations. *Int. J. Pharm.* 182 41–47. 10.1016/s0378-5173(99)00067-8 10332073

[B13] Doepker C. Lieberman H. R. Smith A. P. Peck J. D. El-Sohemy A. Welsh B. T. (2016). Caffeine: friend or foe? *Annu. Rev. Food Sci. Technol.* 7 117–137. 10.1146/annurev-food-041715-033243 26735800

[B14] Dondi D. Albini A. Serpone N. (2006). Interactions between different solar UVB/UVA filters contained in commercial suncreams and consequent loss of UV protection. *Photochem. Photobiol. Sci.* 5 835–843. 10.1039/b606768a 17047836

[B15] Food and Drug Administration [FDA] (2011). *Department of Health and Human Services. 21 CRF parts 201 and 310. Labeling and Effectiveness Testing: Sunscreen Drug Products for Over-The-Counter Human Use — Small Entity Compliance Guide; Final Rule*, Vol. 76 Silver Spring, MD: Food and Drug Administration, 46.

[B16] Hakooz N. M. (2009). Caffeine metabolic ratios for the in vivo evaluation of CYP1A2, N-acetyltransferase 2, xanthine oxidase and CYP2A6 enzymatic activities. *Curr. Drug Metab.* 10 329–338. 10.2174/138920009788499003 19519341

[B17] Herman A. Herman A. P. (2013). Caffeine’s mechanisms of action and its cosmetic use. *Skin Pharmacol. Physiol.* 2013 8–14. 10.1159/000343174 23075568

[B18] Horbury M. D. Baker L. A. Quan W. D. Greenough S. E. Stavros V. G. (2016). Photodynamics of potent antioxidants: ferulic and caffeic acids. *Phys. Chem. Chem. Phys.* 18 17691–17697. 10.1039/c6cp01595f 27310931

[B19] Hu T. Khambatta Z. S. Hayden P. J. Bolmarcich J. Binder R. L. Robinson M. K. (2010). Xenobiotic metabolism gene expression in the EpiDermin vitro 3D human epidermis model compared to human skin. *Toxicol. In Vitro* 24 1450–1463. 10.1016/j.tiv.2010.03.013 20350595

[B20] International Agency for Research on Cancer [IARC] (2012). *A Review of Human Carcinogens. Radiation. IARC Monographs on the Evaluation of Carcinogenic Risks to Humans* Vol. D. Lyon: International Agency for Research on Cancer. PMC7681469 1683674

[B21] Jacobs L. E. Weavers L. K. Houtz E. F. Chin Y. P. (2012). Photosensitized degradation of caffeine: role of fulvic acids and nitrate. *Chemosphere* 86 124–129. 10.1016/j.chemosphere.2011.09.052 22055309

[B22] Johnson S. Moss G. Thomas C. P. (2006). In vitro transdermal delivery of caffeine, theobromine, theophylline and catechin from extract of Guarana, Paullinia Cupana. *Int. J. Pharm.* 317 26–31. 10.1016/j.ijpharm.2006.02.042 16600539

[B23] Karsili T. N. Marchetti B. Ashfold M. N. Domcke W. (2014). Ab initio study of potential ultrafast internal conversion routes in oxybenzone, caffeic acid, and ferulic acid: implications for sunscreens. *J. Phys. Chem. A* 118 11999–12010. 10.1021/jp507282d 25137024

[B24] Kawasumi M. Lemos B. Bradner J. E. Thibodeau R. Kim Y. S. Schmidt M. (2011). Protection from UV-induced skin carcinogenesis by genetic inhibition of the ataxia telangiectasia and Rad3-related (ATR) kinase. *Proc. Natl. Acad. Sci. U.S.A.* 108 13716–13721. 10.1073/pnas.1111378108 21844338 PMC3158235

[B25] Klan P. Wirz J. (2009). *Photochemistry of Organic Compounds: From Concepts to Practice.* Chichester: Wiley, 10.1002/9781444300017

[B26] Lee C. (2000). Antioxidant ability of caffeine and its metabolites based on the study of oxygen radical absorbing capacity and inhibition of LDL peroxidation. *Clin. Chim. Acta* 295 141–154. 10.1016/s0009-8981(00)00201-1 10767400

[B27] León-Carmona J. R. Galano A. (2011a). Is caffeine a good scavenger of oxygenated free radicals?. *J. Phys. Chem. B* 115 4538–4546. 10.1021/jp201383y 21438616

[B28] León-Carmona J. R. Galano A. (2011b). Uric and 1-methyluric acids: metabolic wastes or antiradical protectors? *J. Phys. Chem. B* 115 15430–15438. 10.1021/jp209776x 22097927

[B29] Lu J. Lin P. H. Yao Q. Chen C. (2010). Chemical and molecular mechanisms of antioxidants: experimental approaches and model systems. *J. Cell. Mol. Med.* 14 840–860. 10.1111/j.1582-4934.2009.00897.x 19754673 PMC2927345

[B30] Lu Y. P. Lou Y. R. Liao J. Xie J. G. Peng Q. Y. Yang C. S. (2005). Administration of green tea or caffeine enhances the disappearance of UVB-induced patches of mutant p53 positive epidermal cells in SKH-1 mice. *Carcinogenesis* 26 1465–1472. 10.1093/carcin/bgi086 15817611

[B31] Lu Y. P. Lou Y. R. Xie J. G. Peng Q. Y. Liao J. Yang C. S. (2002). Topical applications of caffeine or (–)-epigallocatechin gallate (EGCG) inhibit carcinogenesis and selectively increase apoptosis in UVB-induced skin tumors in mice. *Proc. Natl. Acad. Sci. U.S.A.* 99 12455–12460. 10.1073/pnas.182429899 12205293 PMC129466

[B32] Lu Y. P. Lou Y. R. Xie J. G. Peng Q. Y. Zhou S. Lin Y. (2007). Caffeine and caffeine sodium benzoate have a sunscreen effect, enhance UVB-induced apoptosis, and inhibit UVB-induced skin carcinogenesis in SKH-1 mice. *Carcinogenesis* 28 199–206. 10.1093/carcin/bgl112 16864596

[B33] Luu-The V. Duche D. Ferraris C. Meunier J. R. Leclaire J. Labrie F. (2009). Expression profiles of phases 1 and 2 metabolizing enzymes in human skin and the reconstructed skin models Episkin and full thickness model from Episkin. *J. Steroid Biochem. Mol. Biol.* 116 178–186. 10.1016/j.jsbmb.2009.05.011 19482084

[B34] Martini D. Del Bo’ C. Tassotti M. Riso P. Del Rio D. Brighenti F. (2016). Coffee consumption and oxidative stress: a review of human intervention studies. *Molecules* 21:979. 10.3390/molecules21080979 27483219 PMC6274123

[B35] Masnec I. S. Kotrulja L. Situm M. Poduje S. (2010). New option in photoprotection. *Coll. Antropol.* 34 257–262. 21302729

[B36] Nawrot P. Jordan S. Eastwood J. Rotstein J. Hugenholtz A. Feeley M. (2003). Effects of caffeine on human health. *Food Addit. Contam.* 20 1–30. 10.1080/0265203021000007840 12519715

[B37] Oliveira C. A. Dario M. F. Sarruf F. D. Mariz I. F. Velasco M. V. Rosado C. (2016). Safety and efficacy evaluation of gelatin-based nanoparticles associated with UV filters. *Colloids Surf. B Biointerfaces* 140 531–537. 10.1016/j.colsurfb.2015.11.031 26613861

[B38] Oliveira C. A. Peres D. D. Rugno C. M. Kojima M. Pinto C. A. Consiglieri V. O. (2015). Functional photostability and cutaneous compatibility of bioactive UVA sun care products. *J. Photochem. Photobiol. B Biol.* 148 154–159. 10.1016/j.jphotobiol.2015.04.007 25920069

[B39] Peres D. A. de Oliveira C. A. da Costa M. S. Tokunaga V. K. Mota J. P. Rosado C. (2016). Rutin increases critical wavelength of systems containing a single UV filter and with good skin compatibility. *Skin Res. Technol.* 22 325–333. 10.1111/srt.12265 26346940

[B40] Peres D. D. Ariede M. B. Candido T. M. de Almeida T. S. Lourenço F. R. Consiglieri V. O. (2017). Quality by design (QbD), process analytical technology (PAT), and design of experiment applied to the development of multifunctional sunscreens. *Drug Dev. Ind. Pharm.* 43 246–256. 10.1080/03639045.2016.1236809 27627681

[B41] Peres D. D. Sarruf F. D. de Oliveira C. A. Velasco M. V. R. Baby A. R. (2018). Ferulic acid photoprotective properties in association with UV filters: multifunctional sunscreen with improved SPF and UVA-PF. *J. Photochem. Photobiol. B Biol.* 185 46–49. 10.1016/j.jphotobiol.2018.05.026 29864725

[B42] Piérard G. E. (1998). EEMCO guidance for the assessment of skin colour. *J. Eur. Acad. Dermatol. Venereol.* 10 1–11. 10.1111/j.1468-3083.1998.tb00921.x 9552751

[B43] Pinnagoda J. Tupkek R. Agner T. Serup J. (1990). Guidelines for transepidermal water loss (TEWL) measurement. *Contact Dermatitis* 22 164–178. 10.1111/j.1600-0536.1990.tb01553.x 2335090

[B44] Rodrigues F. Alves A. C. Nunes C. Sarmento B. Amaral M. H. Reis S. (2016). Permeation of topically applied caffeine from a food by product in cosmetic formulations: is nanoscale in vitro approach an option? *Int. J. Pharm.* 513 496–503. 10.1016/j.ijpharm.2016.09.059 27662805

[B45] Satellite Division and Environmental Systems [DSA] (2014). *Ultraviolet Index.* Available at: http://satelite.cptec.inpe.br/acervo/acervo.formulario.logic (accessed May 22, 2017)

[B46] Scurachio R. S. Mattiucci F. Santos W. G. Skibsted L. H. Cardoso D. R. (2016). Caffeine metabolites not caffeine protect against riboflavin photosensitized oxidative damage related to skin and eye health. *J. Photochem. Photobiol. B Biol.* 163 277–283. 10.1016/j.jphotobiol.2016.08.042 27611451

[B47] Shalbaf M. Gibbons N. C. Wood J. M. Maitland D. J. Rokos H. Elwary S. M. (2008). Presence of epidermal allantoin further supports oxidative stress in vitiligo. *Exp. Dermatol.* 17 761–770. 10.1111/j.1600-0625.2008.00697.x 18328088

[B48] Shi X. Dalal N. S. Jain A. C. (1991). Antioxidant behavior of caffeine: efficient scavenging of hydroxyl radicals. *Food Chem. Toxicol.* 29 1–6. 10.1016/0278-6915(91)90056-D 1847890

[B49] Skin Cancer Foundation (2016). Available at: http://www.skincancer.org/skin-cancer-information (accessed April 01, 2017).

[B50] Szeto Y. T. Tong H. H. (2010). Caffeine as a photoprotective agent for diminishing phototoxicity. *Toxicol. Ind. Health* 26 667–670. 10.1177/0748233710375947 20630985

[B51] Tan E. M. M. Hilbers M. Buma W. J. (2014). Excited-state dynamics of isolated and microsolvated cinnamate-based UV-B sunscreens. *J. Phys. Chem. Lett.* 5 2464–2468. 10.1021/jz501140b 26277816

[B52] Tomazelli L. C. Ramos M. M. A. Sauce R. Cândido T. M. Sarruf F. D. Pinto C. A. S. O. (2018). SPF enhancement provided by rutin in a multifunctional sunscreen. *Int. J. Pharm.* 552 401–406. 10.1016/j.ijpharm.2018.10.015 30308277

[B53] Turro N. J. Ramamurthy V. Scaiano J. C. (2010). *Modern Molecular Photochemistry of Organic Molecules.* Sausalito, CA: University Science Books, 10.1021/ja1036176

[B54] van de Sandt J. J. van Burgsteden J. A. Cage S. Carmichael P. L. Dick I. Kenyon S. (2002). In vitro predictions of skin absorption of caffeine, testosterone, and benzoic acid: a multi-centre comparison study. *Regul. Toxicol. Pharm.* 39 271–281. 10.1016/j.yrtph.2004.02.004 15135208

[B55] Velasco M. V. Sarruf F. D. Salgado-Santos I. M. Haroutiounian-Filho C. A. Kaneko T. M. Baby A. R. (2008). Broad spectrum bioactive sunscreens. *Int. J. Pharm.* 363 50–57. 10.1016/j.ijpharm.2008.06.031 18662760

[B56] Zesch A. Schaefer H. Stuttgen G. (1979). The quantitative distribution of percutaneously applied caffeine in the human skin. *Arch. Dermatol. Res.* 266 277–283. 10.1007/bf00418573 526050

